# Long-Term Trends in Shooting Performance in the NBA: An Analysis of Two- and Three-Point Shooting across 40 Consecutive Seasons

**DOI:** 10.3390/ijerph20031924

**Published:** 2023-01-20

**Authors:** Tomasz Zając, Kazimierz Mikołajec, Paweł Chmura, Marek Konefał, Michał Krzysztofik, Piotr Makar

**Affiliations:** 1 Human Performance Laboratory, The Jerzy Kukuczka Academy of Physical Education in Katowice, Mikołowska 72A, 40-065 Katowice, Poland; 2 Department of Basketball and Football, The Jerzy Kukuczka Academy of Physical Education in Katowice, Mikołowska 72A, 40-065 Katowice, Poland; 3 Department of Team Games, Wrocław University of Health and Sport Sciences, I.J., Paderewskiego 35, 51-612 Wrocław, Poland; 4 Department of Biological and Motor Sport Bases, Wrocław University of Health and Sport Sciences, I.J., Paderewskiego 35, 51-612 Wrocław, Poland; 5 Institute of Sport Sciences, The Jerzy Kukuczka Academy of Physical Education in Katowice, 40-065 Katowice, Poland; 6 Faculty of Physical Education, Gdańsk University of Physical Education and Sport, 80-336 Gdańsk, Poland

**Keywords:** basketball, trend analysis, game outcomes, field goals

## Abstract

This study aims to depict two-point and three-point shooting trends and explore their influence on game outcomes in the NBA across 40 consecutive seasons. Therefore, the following game-related statistics were considered: total points per game (PPG), games played (GP), field goals made (FGM), field goal attempts (FGA), field goal percentage (FG%), two-point field goals made (2PM), two-point field goal attempts (2PA), two-point field goal percentage (2P%), three-point field goals made (3PM), three-point field goal attempts (3PA), three-point field goal percentage (3P%), and a three-point field goal to two-point field goal ratio (3P/2P). The fixed-base indexes and inter-decade ANOVAs or Friedman tests were used as the main statistical tools. The number of 3PA significantly increased over time, while the number of 2PA decreased. A significant increase in 3P% was also observed, whereas 2P% remained relatively stable over the analyzed period. This study also revealed a higher number of ball possessions and more points scored per game, especially in the last decade of NBA competition.

## 1. Introduction

Basketball has gone through several changes since its invention by James Naismith in 1896. We evidence the foundation of the BBA (Basketball Association of America) and its fusion with the NBL (National Basketball League), finally forming the National Basketball Association (NBA) in 1976. Since the inception of the NBA, there has been a constant evolution in game regulations, resulting in one of the most popular sports disciplines in the world. One of the most significant changes in game regulations in the recent history of the NBA refers to the inclusion of the three-point line.

To succeed in the NBA, teams and players must show game-changing abilities that allow them to adapt to the demands of the game in terms of pace and defensive pressure (i.e., blocks, steals), which denotes the increased influence of athleticism [1]. In addition, top-level players have adapted their style of play by progressively including the three-point shot into their offensive arsenal. This tendency is further supported by the fact that the number of three-point shots attempted by a team per game (3PA) has drastically increased over the years. In 1979, when the three-point line was first established in the NBA, the average number of 3PA per game was just 2.8 (Table 1). Conversely, in the 2018–2019 season, that number increased to 32.0, which amounted to an increase of over 1000% (basketball-reference.com, accessed on 1 July 2019). This tendency was followed by a significant improvement in three-point field goal efficacy (3P%), from a low 28% during the initial season of the three-point shot in the NBA to an approximate level of 35–36% in the last few seasons [2].

According to Goldsberry [3], a new shot efficiency landscape has emerged in the last few years in the NBA. Through the use of spatial–temporal data of players’ shooting actions, combined with top-end computational methods, it became possible to disclose these arising patterns of behavior. Considering the average points per field goal attempt within the 2013/2014 to 2017/2018 period, two different shooting efficacy patterns can be identified. While two-point shots from a middle distance are associated with 0.85 to 0.90 points per shot, three-point shots and shots close to the basket represent, respectively, nearly 1.20 and 1.10 points per shot [3]. The best NBA perimeter players are converting over 40% of their long-distance shots, which corresponds to an efficacy of 60% in two-point field goals. Recently, a study by Esteves et al. [4] highlighted the importance of distance shooting in the game as the three-point misses and three-point attempts stood out as the discriminators of fixture congestion cycles. Therefore, new shooting patterns have been observed in NBA players, concentrating on shooting opportunities in proximal areas to the basket or by distance shots (i.e., three-point shots). However, more research is needed to better understand the evolution of two-point and three-point shooting trends over time in the NBA.

Going beyond the individual player contributions, the cumulative effect at the team level of such shooting effectiveness may also greatly contribute to winning the games [5,6]. Shooting efficacy has been reported as one of the key game performance variables that impact game performance in the NBA [7]. Teams with a higher effective field goal percentage (eFG%) than their opponents tend to win around 81% of their games during the regular season, and their winning efficiency increases to 90% in the playoffs [8]. Therefore, the possibility of expanding the knowledge on two-point and three-point shooting trends concerning collective efficacy indicators would allow a better understanding of how these variables combined together can impact game performance.

From a practical perspective, the observed change in shooting patterns entails an intentional adaptation of both coaches and athletes to better explore three-point shot and layup (i.e., shooting in proximal areas to the basket) opportunities while compromising midrange jump shots. This means that to optimize game strategy and enhance winning possibilities, the coaching staff must critically manage the right proportion of two- and three-point shots, according to the characteristics of the team’s roster and the strategy to approach the upcoming game [9]. Since the level of talent across NBA teams can vary, coaching staff try to get the most out of the available players on the roster. The importance of roster management has been highlighted by previous research, for instance, when exploring the effects of substitutions on scoring performances over the course of a game [10]. There are additional variables that critically affect player performance profiles and, in turn, impact the in-game decisions made by coaching staff, such as the phase of the competition [11], injury risk [12], and quality of the opposition [13]. Considering the introduction of advanced statistical analysis, coaching staff may monitor game dynamics, which allows for more appropriate decisions and increased possibilities of winning. Game indicators are, then, valuable tools for coaching staff to promote specific shooting trends, both from an individual and a collective point of view [14].

To the best of our knowledge, there is limited research on the evolution of two-point and three-point shooting trends concerning game outcomes in the NBA. Considering the above, the main objective of this study was to identify and describe the three-point and two-point shooting trends and their respective impact on game outcomes in the NBA during 4 consecutive decades. It is important to note that the selection of this specific time period was considered to match the introduction of the three-point line in the NBA in 1979. We analyzed the differences in three-point field goal attempts and their efficacy compared with the two-point shots throughout the last 40 NBA seasons. We also attempted to understand how shooting trends have changed and how they impact game outcomes. Our main hypothesis states that the number of three-point shot attempts constantly increases as their efficacy has a more significant impact on the game outcome.

## 2. Materials and Methods

Data from current and archival seasons were obtained from the open-access NBA records site (www.basketball-reference.com (accessed on 1 July 2019)). These statistical databases have been deemed reliable by previous research [15,16].

The records contained a total of 40 seasons (one was excluded because of a league lockout). The data were collected from the 1979–1980 season, when the 3-point shot was first included in game regulations, to the 2018–2019 season. The analyzed variables were as follows: total points per game (PPG), games played (GP), field goals made (FGM), field goal attempts (FGA), field goal percentage (FG%), 2-point field goals made (2PM), 2-point field goal attempts (2PA), 2-point field goal percentage (2P%), 3-point field goals made (3PM), 3-point field goal attempts (3PA), 3-point field goal percentage (3P%), and 3-point field goal to 2-point field goal ratio (3P/2P).

### Statistical Analysis

All variables were expressed as mean ± standard deviation (SD). The normality assumption was verified using the Kolmogorov–Smirnov test. The numbers of quality data for analyzing groups were obtained using the analysis of the contingency table.

The one-way repeated-measures ANOVA or Friedman test (in case of violated data distribution) was used with a significance set at *p* < 0.05 to determine differences between 2PA, 2PM, 2P% and 3PA, 3PM, 3P% as well as inter-decade PPG and FG%. When appropriate, post hoc tests with Bonferroni correction were used to analyze the pairwise comparisons.

To determine the trends of changes, fixed-base indexes were used based on time series, and results were presented in percentage form.

To make the results and respective changes more transparent, they were divided into four decades (1st D—first decade, from 1979–1988; 2nd D—second decade, from 1989–1999; 3rd D—third decade, from 2000–2009; 4th D—fourth decade, from 2010–2019). The remaining analyses were performed using SPSS (version 25.0; SPSS, Inc., Chicago, IL, USA).

## 3. Results

### 3.1. Analysis of Two-Point and Three-Point Field Goals Attempted and Made

**Figure 1:**
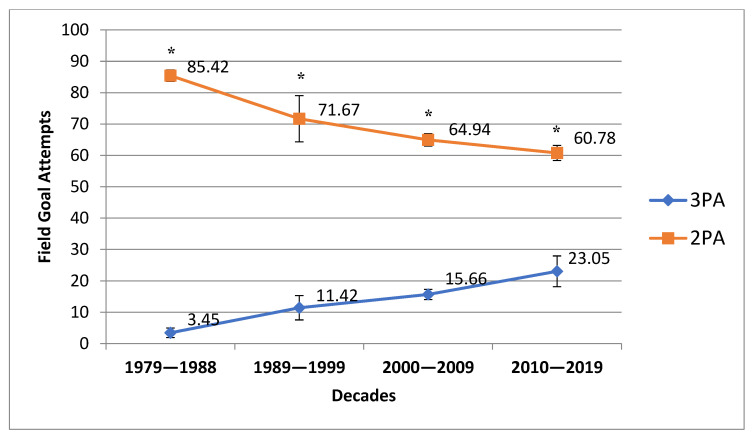
Three-point and two-point field goal attempt trends over the last 40 NBA seasons. * Statistically significant compared with the previous decade with *p* < 0.05.

**Figure 2:**
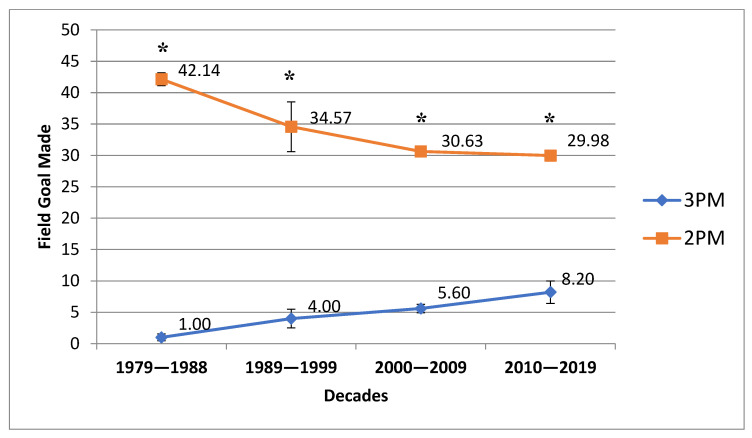
Trends in three-point and two-point field goals made over the last 40 NBA seasons. * Statistically significant compared with the previous decade with *p* < 0.05.

Table 1 presents descriptive data of analyzed variables. During the last 40 NBA seasons, from 1979 to 2019, the number of 3PA has significantly increased between each decade, from 3.45 to 23.05 per game (Figure 1 and Table 2). Conversely, the number of 2PA has significantly decreased from the 1980s to the modern era of the NBA, from 85.42 to 60.78 per game (Figure 1 and Table 2). Similar trends were found in the cases of 3PM and 2PM, respectively (Figure 2 and Table 2).

One-way repeated-measures ANOVA revealed statistically significant differences between the analyzed decades for the 2PA and 3PA, 3PM variables. Similarly, the Friedman test was used for 2PM. Table 2 presents the results of the ANOVA and Friedman tests and post hoc comparisons between decades for the mentioned variables.

### 3.2. Total Points per Game, Field Goal Percentage, and Two-Point and Three-Point Field Goal Percentage Analysis

Table 3 presents the results of the Friedman tests and post hoc comparisons between decades for FG%, 2P%, and 3P%.

**Figure 3:**
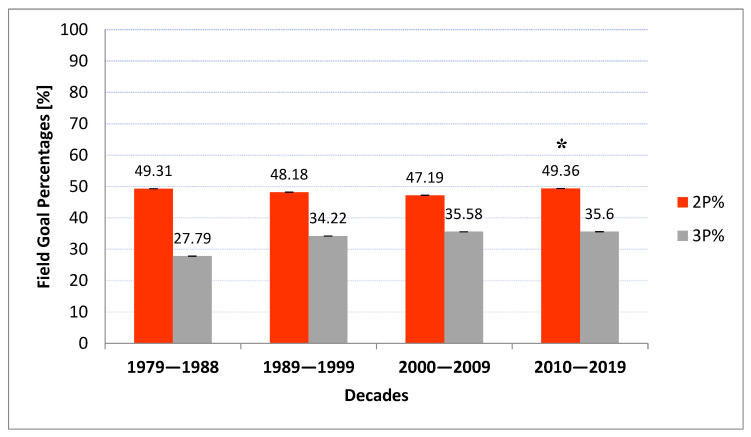
Three-point and two-point field goal percentages over four consecutive NBA decades. * Statistically significant compared with the previous decade with *p* < 0.05.

Inter-decade fixed-base indexes remained relatively stable over this time (1st D—49.3%, 2nd D—48.2%, 3rd D—47.2%, and 4th D—49.4%), whereas the 3P% exhibited an increasing trend for its mean (1st D—27.8%, 2nd D—34.2%, and 3rd D and 4th D—35.6%) (Figure 3). From 1992/1993 onwards, the percentage of three-point field goals made did not fall under 33.3% (Table 1). The average 3P% analyzed using fixed-base indexes between the second and fourth decades varied between 34.2% and 35.6% despite a significant increase in 3PA over each following year (starting from 11.42 to 23.05 3PA per game in the decade ending in 2018/2019) (Figure 1). On the other hand, the 2P% demonstrated by fixed-base indexes remained relatively stable in the 2nd D and 4th D decades (48.2% to 49.4%), despite a huge decrease in the number of attempts (which dropped from 71.7 to 60.8 2PA per game) (Figure 1). This means that the probability of a successful three-point shot remained higher than the probability of a successful two-point shot for each analyzed NBA decade.

**Figure 4:**
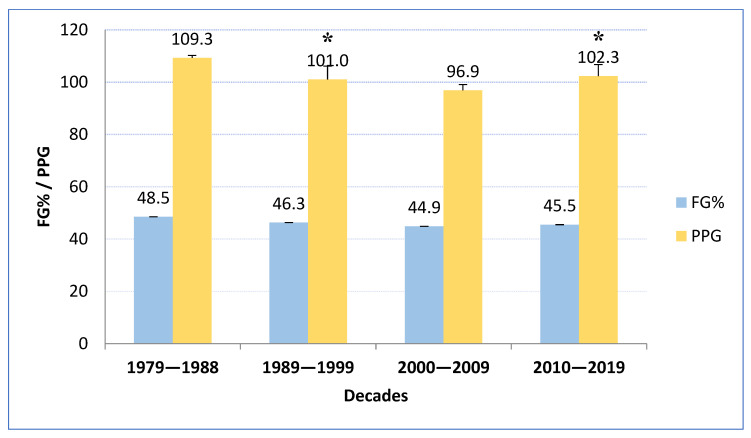
Total field goal percentages compared with total points scored per game over four consecutive NBA decades. * Statistically significant compared with the previous decade with *p* < 0.05.

The analysis with fixed-base indexes revealed that in the early years of introducing the three-point shot, the effectiveness of all field goals in the first decade reached 48.5% (FG%), expressing an average of 109.3 PPG. In each subsequent decade, analysis with fixed-base indexes revealed that there was a decrease in the effectiveness of all field goals (2nd D—46.3%, 3rd D—44.9%), which also translated into smaller numbers of PPG (2nd D—101.0, 3rd D—96.9 PPG). Only in recent years has this indicator changed, and in both cases, there has been an increasing trend. The efficacy improved up to 45.5% and the average number of points scored per game increased by almost five points (4th D—102.3 PPG) (Figure 4).

## 4. Discussion

The game of professional basketball has changed significantly over the past 4 analyzed decades. The new trends are most visible in the NBA, which is the world’s leading basketball league, annually recruiting talented and skilled athletes to the 30 teams that compete at the highest sports level. The purpose of this study was to provide an analysis of the three-point versus the two-point shooting trends and how the game outcome has been affected by rule and game structure changes in the NBA since the 1979/80 season. The considered data included both field goal attempts as well as shooting efficacy. We also attempted to present the trends related to the number of ball possessions and the total number of scored points per game as factors related to shooting performance.

The results clearly indicate that during the last 40 NBA seasons (from 1979 to 2019), the number of 3PA has significantly increased. On the other hand, 2PA, and especially the midrange jump shot frequency, have significantly decreased. We also recorded a significant increase in 3PA over each following year from 2.8 to 32.0 per game in the last considered season of 2018/2019. Both previously mentioned trends for 2PA and 3PA are statistically significant (Table 1). The increase in 3PA is related to the fact that most of the NBA teams play significantly more behind the three-point line than previously. This game strategy is possible because of the significant improvement in long-distance shooting skills, not only of perimeter players but also of post players. One of the fundamental reasons for this phenomenon is that modern training methods have improved all-around skills that in the past decades were destined only for certain basketball positions (PG, SG, SF, PF, C). The physical condition of players, as well as agility, speed, explosiveness, and athleticism impact their ability to play at several positions. The development of long-distance shooting skills and techniques also has some important in-game benefits. For example, these abilities create space for frontcourt players to operate under the basket, open up the lane for backcourt players to drive to the basket and execute the defense that allows too much space for shooters, or simply allow a player to get back on transition defense.

From a mathematical point of view, the points scored from under the basket have a very high average efficacy of 65%, which translates to 1.30 points per shot. Unfortunately, when we explore two-point shots taken within 2–5 m away from the basket, the shooting efficacy falls to around 38%, which gives just 0.76 points per shot [6]. This indicates that the efficacy of three-point and two-point midrange shots is very similar. Therefore, in terms of appropriate shot selection, a team’s priority should be creating wide-open 2PA after a drive and 3PA performed by highly skilled players. The two-point midrange jump shot seems to be the least effective and least desired skill in the NBA today. If we analyze the tactical aspects of a strong three-point offense, we can observe that it opens other offensive options related to penetration through better spacing on the court. Effective offensive inside play forces the post players to focus on inside defense, which opens the floor for three-point shots. These game tactics are quite visible in the NBA and have a significant impact on game-related statistics.

The efficacy of both the mean and variance of the probability of a successful two-point shot have remained relatively stable over the last four decades (1st D—49.3%, 2nd D—48.2%, 3rd D—47.2%, and 4th D—49.4%). On the contrary, the probability of a successful three-point shot has exhibited an increasing trend for its mean and a decreasing trend for its variance: 1st D—27.8%, 2nd D—34.2%, 3rd D—35.6%, and 4th D—35.6%. Both presented trends are statistically significant (Figure 2). These results suggest that coaches and players perceived shot selection as one of the most important aspects of success in basketball due to its direct influence on the total amount of scored points and game outcome. Considering the improved efficacy of the three-point shot, coaching staff have steadily increased the number of long-distance shots, considering the benefits of scoring more points.

Shooting effectiveness is the most significant element of team success in professional basketball. Successful shooting performance depends on many factors but generally represents the level of individual and collective skills [17,18]. It appears that the positive tendency in three-point shooting accuracy is related to the revolution in tactics—“position-less basketball”. The typical on-court positions, such as the point guard, shooting guard, small forward, power forward, and center, have been replaced by versatile players who can play several positions. Typical post players are now capable of shooting the three-point shot with great success. Today, players in the NBA have new roles, use different spacing and ball movements, and create shooting opportunities in a different way. The emergence of new positions in the NBA, such as the “point-forward,” “stretch four,” and even the “stretch five” has played a relevant role in the evolution of the game and the three-point shot. Some of the best post players, such as Lopez, Griffin, Porzingis, Jokić, Aldridge, and Davis, have significantly improved their long-distance shooting technique and efficacy and now successfully use this technique as an additional offensive threat. The number of long-range shots taken by post players increased from 4000 in the 2012/13 season to more than 10,000 in the 2017/18 season. This indicates an increase of over 150% in just five years. Therefore, we observed a decrease in field goal effectiveness in each subsequent decade (2nd D—46.3%, 3rd D—44.9%), and, consequently, fewer points scored per game (2nd D—101.0, 3rd D—96.9). However, in recent years efficacy has improved up to 45.5%, and the average number of points scored per game has increased by almost five points: 4th D—102.3. The last considered regular season, 2018/19, also shows very high three-point shooting efficacy and a significant contribution to total points scored by top teams such as the Rockets, Bucks, Warriors, and Raptors. On the other hand, the statistical analysis indicates that the teams with the worst NBA record reached the lowest three-point shot field goal percentages and much fewer three-point field goals made per game (Suns, Lakers, Knicks, Mavericks, Wizards, and Grizzlies). These findings confirm how the modern concept of play, defined as position-less basketball supported by the three-point shot explosion, has affected the style of play in the NBA. Several studies have confirmed that winning teams achieve better shooting efficacy [19,20,21], and they emphasize the importance of three-point shot efficacy in discriminating between winning and losing teams [10].

The data analysis also showed a relevant increase in the number of ball possessions in the last decade (1st D—101.6, 2nd D—93.9, 3rd D—91.4, and 4th D—94.5) and the number of scored points per game (1st D—109.3, 2nd D—101.0, 3rd D—96.9, and 4th D—102.3) as a result of improved offensive play. Trninić et al. [22] and Sampaio and Janeira [23] concluded that the higher number of ball possessions creates more possibilities for offensive actions. It can be hypothesized that the increment in ball possessions is related to greater team mobility and capacity to play up-tempo transition offenses. We can also observe a tendency to shorten particular plays due to rule changes and the determination to create quick, open shots or fast break situations. The number of scored points per game increased because of the higher number of shots taken and better shooting efficacy, especially for the three-point shots. The game-related variables under consideration were also affected by the increased number of ball possessions observed in NBA games. The NBA player’s performance can be explained not only by a higher level of technical skill but also by a more developed perception [13,24].

### 4.1. Limitations

Despite the results obtained in this study, we were mostly exploring the changes in shooting trends, which only represent the offensive part of game evolution. Therefore, the three-point shooting issue requires further analysis, particularly focused on its impact on winning basketball games, its effectiveness going forward, or simply finding a solution in game adjustments to prevent further three-point field goal population growth. In addition, it is important to continue research based not only on regular NBA season games but also on the elimination round, where defensive strategies presumably play a more significant role.

### 4.2. Practical Application

These new trends in basketball seem to be significant issues for further analysis, as they determine success in the most prominent basketball league in the world and could be a key factor in improving game outcomes. A number of factors, including deeper knowledge of analytics, access to comprehensive data, play-by-play game feedback, etc., allow coaching staff and general managers to fully evaluate team needs and weaknesses. Knowing which adjustments can be incorporated to increase the chances of winning basketball games could also lead to crucial reversal changes in future shooting trends.

## 5. Conclusions

To conclude, our study captures the evolution of three- and two-point shooting performance in the NBA from 1979 to 2019.

The results revealed that the number of 3PA significantly increased while the number of 2PA decreased. Furthermore, a significant increase was observed in the case of three-point shooting efficacy, whereas two-point shooting has remained relatively stable. All recorded changes are related to the new approach to offensive team tactics defined as “position-less basketball”, as well as the noticeable development of individual shooting skills, especially long-distance shooting skills. Considering that most technical and tactical trends are derived from the NBA, the above findings should be analyzed and adopted by basketball specialists worldwide.

Three-point shooting revolutionized the game of basketball, and it is becoming harder for coaches and players to make proper adjustments during games, especially when opposing teams play at high three-point field goal efficacy. The constant increases in the number of three-point shots made, as well as higher three-point shot effectiveness, have turned basketball into an exceptionally offensive sport. In order to achieve the best game outcomes, coaches need to optimize the right proportion of field goal attempts on the offensive end along with limiting three-point efficacy on the defensive end.

## Figures and Tables

**Table 1:** Descriptive data of selected variables.

Decades	Season	GP [n]	PPG [n]	FGM [n]	FGA [n]	FG% [%]	3PM [n]	3PA [n]	3P% [%]	2PM [n]	2PA [n]	2P% [%]	FTM [n]	FTA [n]	FT [%]	Pace Factor	NBA Title
																(Ball Possessions Per 48’)	
**1D**	1979/80	82	109.3	43.6	90.6	48.1%	0.8	2.8	28.0%	42.9	87.9	48.8%	21.3	27.8	76.4%	103.1	Los Angeles Lakers
	1980/81	82	108.1	43.0	88.4	48.6%	0.5	2.0	24.5%	42.5	86.4	49.1%	21.7	28.9	75.1%	101.8	Boston Celtics
	1981/82	82	108.6	43.3	88.2	49.1%	0.6	2.3	26.2%	42.7	86.0	49.7%	21.3	28.6	74.6%	100.9	Los Angeles Lakers
	1982/83	82	108.5	43.5	89.7	48.5%	0.5	2.3	23.8%	43.0	87.4	49.2%	20.9	28.3	74.0%	103.1	Philadelphia 76ers
	1983/84	82	110.1	43.5	88.4	49.2%	0.6	2.4	25.0%	42.9	86.0	49.9%	22.6	29.7	76.0%	101.4	Boston Celtics
	1984/85	82	110.8	43.8	89.1	49.1%	0.9	3.1	28.2%	42.9	86.0	49.9%	22.4	29.4	76.4%	102.1	Los Angeles Lakers
	1985/86	82	110.2	43.2	88.6	48.7%	0.9	3.3	28.2%	42.3	85.3	49.5%	22.9	30.3	75.6%	102.1	Boston Celtics
	1986/87	82	109.9	42.6	88.8	48.0%	1.4	4.7	30.1%	41.2	84.1	49.0%	23.2	30.5	76.3%	100.8	Los Angeles Lakers
	1987/88	82	108.2	42.1	87.7	48.0%	1.6	5.0	31.6%	40.6	82.7	49.0%	22.3	29.1	76.6%	99.6	Los Angeles Lakers
	1988/89	82	109.2	42.5	89.0	47.7%	2.1	6.6	32.3%	40.4	82.4	49.0%	22.1	28.8	76.8%	100.6	Detroit Pistons
**Mean**		82	109.3	43.1	88.9	48.5%	1.0	3.5	27.8%	42.1	85.4	49.3%	22.1	29.1	75.8%	101.6	
**SD**		0.0	0.9	0.6	0.8	0.0	0.5	1.5	0.0	1.0	1.8	0.0	0.8	0.9	0.0	1.1	
**2D**	1989/90	82	107.0	41.5	87.2	47.6%	2.2	6.6	33.1%	39.3	80.6	48.8%	21.8	28.5	76.4%	98.3	Detroit Pistons
	1990/91	82	106.3	41.4	87.2	47.4%	2.3	7.1	32.0%	39.1	80.1	48.8%	21.3	27.9	76.5%	97.8	Chicago Bulls
	1991/92	82	105.3	41.3	87.3	47.2%	2.5	7.6	33.1%	38.7	79.7	48.6%	20.2	26.7	75.9%	96.6	Chicago Bulls
	1992/93	82	105.3	40.7	86.0	47.3%	3.0	9.0	33.6%	37.7	77.0	48.9%	20.9	27.7	75.4%	96.8	Chicago Bulls
	1993/94	82	101.5	39.3	84.4	46.6%	3.3	9.9	33.3%	36.0	74.5	48.3%	19.6	26.6	73.4%	95.1	Houston Rockets
	1994/95	82	101.4	38.0	81.5	46.6%	5.5	15.3	35.9%	32.5	66.2	49.1%	19.9	27.1	73.7%	92.9	Houston Rockets
	1995/96	82	99.5	37.0	80.2	46.2%	5.9	16.0	36.7%	31.2	64.1	48.6%	19.5	26.4	74.0%	91.8	Chicago Bulls
	1996/97	82	96.9	36.1	79.3	45.5%	6.0	16.8	36.0%	30.0	62.5	48.0%	18.7	25.3	73.8%	90.1	Chicago Bulls
	1997/98	82	95.6	35.9	79.7	45.0%	4.4	12.7	34.6%	31.5	67.0	47.0%	19.4	26.3	73.7%	90.3	Chicago Bulls
	1998/99	50	91.6	34.2	78.2	43.7%	4.5	13.2	33.9%	29.7	65.0	45.7%	18.8	25.8	72.8%	88.9	San Antonio Spurs
**Mean**		79	101.0	38.5	83.1	46.3%	4.0	11.4	34.2%	34.6	71.7	48.2%	20.0	26.8	74.6%	93.9	
**SD**		10.1	5.1	2.7	3.7	0.0	1.5	3.9	0.0	4.0	7.4	0.0	1.0	1.0	0.0	3.5	
**3D**	1999/00	82	97.5	36.8	82.1	44.9%	4.8	13.7	35.3%	32.0	68.4	46.8%	19.0	25.3	75.0%	93.1	Los Angeles Lakers
	2000/01	82	94.8	35.7	80.6	44.3%	4.8	13.7	35.4%	30.8	66.9	46.1%	18.6	24.9	74.8%	91.3	Los Angeles Lakers
	2001/02	82	95.5	36.2	81.3	44.5%	5.2	14.7	35.4%	31.0	66.5	46.5%	17.9	23.8	75.2%	90.7	Los Angeles Lakers
	2002/03	82	95.1	35.7	80.8	44.2%	5.1	14.7	34.9%	30.6	66.1	46.3%	18.5	24.4	75.8%	91.0	San Antonio Spurs
	2003/04	82	93.4	35.0	79.8	43.9%	5.2	14.9	34.7%	29.8	64.9	46.0%	18.2	24.2	75.2%	90.1	Detroit Pistons
	2004/05	82	97.2	35.9	80.3	44.7%	5.6	15.8	35.6%	30.3	64.6	47.0%	19.7	26.1	75.6%	90.9	San Antonio Spurs
	2005/06	82	97.0	35.8	79.0	45.4%	5.7	16.0	35.8%	30.1	63.0	47.8%	19.6	26.3	74.5%	90.5	Miami Heat
	2006/07	82	98.7	36.5	79.7	45.8%	6.1	16.9	35.8%	30.5	62.8	48.5%	19.6	26.1	75.2%	91.9	San Antonio Spurs
	2007/08	82	99.9	37.3	81.5	45.7%	6.6	18.1	36.2%	30.7	63.4	48.4%	18.8	24.9	75.5%	92.4	Boston Celtics
	2008/09	82	100.0	37.1	80.9	45.9%	6.6	18.1	36.7%	30.5	62.8	48.5%	19.1	24.7	77.1%	91.7	Los Angeles Lakers
**Mean**		82	96.9	36.2	80.6	44.9%	5.6	15.7	35.6%	30.6	64.9	47.2%	18.9	25.1	75.4%	91.4	
**SD**		0.0	2.2	0.7	0.9	0.0	0.7	1.6	0.0	0.6	2.0	0.0	0.6	0.9	0.0	0.9	
**4D**	2009/10	82	100.4	37.7	81.7	46.1%	6.4	18.1	35.5%	31.3	63.6	49.2%	18.6	24.5	75.9%	92.7	Los Angeles Lakers
	2010/11	82	99.6	37.2	81.2	45.9%	6.5	18.0	35.8%	30.8	63.2	48.7%	18.6	24.4	76.3%	92.1	Dallas Mavericks
	2011/12	66	96.3	36.5	81.4	44.8%	6.4	18.4	34.9%	30.1	63.0	47.7%	16.9	22.5	75.2%	91.3	Miami Heat
	2012/13	82	98.1	37.1	82.0	45.3%	7.2	20.0	35.9%	30.0	62.1	48.3%	16.7	22.2	75.3%	92.0	Miami Heat
	2013/14	82	101.0	37.7	83.0	45.4%	7.7	21.5	36.0%	30.0	61.5	48.8%	17.8	23.6	75.6%	93.9	San Antonio Spurs
	2014/15	82	100.0	37.5	83.6	44.9%	7.8	22.4	35.0%	29.7	61.2	48.5%	17.1	22.8	75.0%	93.9	Golden State Warriors
	2015/16	82	102.7	38.2	84.6	45.2%	8.5	24.1	35.4%	29.7	60.5	49.1%	17.7	23.4	75.7%	95.8	Cleveland Cavaliers
	2016/17	82	105.6	39.0	85.4	45.7%	9.7	27.0	35.8%	29.4	58.4	50.3%	17.8	23.1	77.2%	96.4	Golden State Warriors
	2017/18	82	106.3	39.6	86.1	46.0%	10.5	29.0	36.2%	29.1	57.1	51.0%	16.6	21.7	76.7%	97.3	Golden State Warriors
	2018/19	82	111.2	41.1	89.2	46.1%	11.4	32.0	35.5%	29.7	57.2	52.0%	17.7	23.1	76.6%	100.0	Toronto Raptors
**Mean**		80	102.1	38.2	83.8	45.5%	8.2	23.1	35.6%	30.0	60.8	49.4%	17.6	23.1	76.0%	94.5	
**SD**		5.1	4.4	1.4	2.5	0.0	1.8	4.9	0.0	0.6	2.4	0.0	0.7	0.9	0.0	5.1	

Data presented as mean and standard deviation (SD). GP—games played, PPG—total points per game, FGM—field goals made, FGA—field goal attempts, FG%—field goal percentage, 3PM—3-point field goals made, 3PA—3-point field goal attempts, 3P%—3-point field goal percentage, 2PM—2-point field goals made, 2PA—2-point field goal attempts, 2P%—2-point field goal percentage, FTM—free throws made, FTA—free throw attempts, FT%—free throws percentage, NBA - National Basketball Association.

**Table 2:** Results of ANOVAs or Friedman tests and post hoc comparisons between decades for 3PA, 3PM and 2PA, 2PM.

Decades	I	II	III	IV	ANOVA/Friedman
3PA					
I		<0.001	<0.001	<0.001	F = 146,939
II	<0.001		0.007	<0.001	
III	<0.001	0.007		<0.001	<0.001
IV	<0.001	<0.001	<0.001		
2PA					
I		<0.001	<0.001	<0.001	F = 136,740
II	<0.001		0.024	0.001	
III	<0.001	0.024		<0.001	<0.001
IV	<0.001	0.001	<0.001		
3PM					
I		<0.001	<0.001	<0.001	F = 137,720
II	<0.001		0.009	<0.001	
III	<0.001	0.009		<0.001	<0.001
IV	<0.001	<0.001	<0.001		
2PM					
I		0.182	0.004	<0.001	test = 25,745
II	0.182		1	0.044	
III	0.004	1		0.846	<0.001
IV	<0.001	0.044	0.846		

Definitions: 3PA—3-point field goal attempts, 2PA—2-point field goal attempts, 3PM—3-point field goals made, 2PM—2-point field goals made.

**Table 3:** Results of ANOVA or Friedman tests and post hoc comparisons between decades for PPG, FG%, 2P%, and 3P%.

Decades	I	II	III	IV	ANOVA/Friedman
PGG					
I		0.006	<0.001	0.004	F = 15,855
II	0.006		0.556	1	
III	<0.001	0.556		0.002	0.002
IV	0.004	1	0.002		
FG%					
I		0.092	<0.001	0.003	test = 22,067
II	0.092		0.500	1	
III	<0.001	0.500		1	<0.001
IV	0.003	1	1		
2P%					
I		0.340	0.004	1	test = 15,483
II	0.340		0.846	1	
III	0.004	0.846		0.034	0.001
IV	1	1	0.034		
3P%					
I		0.072	0.001	<0.001	test = 22,290
II	0.072		0.995	0.846	
III	0.001	0.995		1	<0.001
IV	<0.001	0.846	1		

Definitions: PPG—total points per game, FG%—field goal percentage, 3P%—3-point field goal percentage, 2P%—2-point field goal percentage.

## Data Availability

The datasets used and analyzed during the current study are available from the corresponding author upon reasonable request.
